# Supplementary material for: Hybrid Models and Biological Model Reduction with PyDSTool
Source: PLoS Comput Biol. 2012 Aug 9;8(8):e1002628. doi: 10.1371/journal.pcbi.1002628 (PMC3415397; doi:10.1371/journal.pcbi.1002628)
Supplement: Text S4 — Complete source code for the PyDSTool package (version 0.88.120504). Includes API documentation and help files linking to web pages. This file is identical to the current public release on Sourceforge.net. (ZIP) [file pcbi.1002628.s004.zip › PyDSTool/html/toc-PyDSTool.Symbolic-module.html]

xml version="1.0" encoding="ascii"?


Symbolic


# Module Symbolic

---

## Classes

Fun  
 Input  
 Par  
 QuantSpec  
 Quantity  
 Var  

\_mathobj

## Functions

Diff  
 DiffStr  

\_eval

\_generate\_subderivatives

\_join\_sublist

\_parse\_func\_deriv

\_propagate\_str\_eval

checkbraces  

dofun

ensureQlist  
 ensureStrArgDict  
 evalMultiRefToken  
 expr2fun  
 findMultiRefs  

getdim

getlftrtD

isMultiDef  
 isMultiDefClash  
 isMultiRef  
 prepJacobian  

processMultiDef

processMultiRef  

resolveSpecTypeCombos

strIfSeq

subs  

whoQ

## Variables

API

API\_class  
 Abs  
 Acos  
 Asin  
 Atan  
 Atan2  
 Betavariate  
 Ceil  
 Choice  

Continuous

Cos  
 Cosh  
 DO\_DEC  
 DO\_POW  
 Degrees  

Discrete

DomainType  
 E  
 Exp  
 Expovariate  
 Fabs  
 Floor  
 Fmod  
 Frexp  
 Gammavariate  
 Gauss  
 Getrandbits  
 Getstate  
 Hypot  

Inf

Jumpahead  

LargestInt32

Ldexp  
 Log  
 Log10  
 Lognormvariate  
 Macheps  
 Max  
 Min  
 Modf  

NAMESEP

NaN

Normalvariate  

ONES

POW\_STR  
 Paretovariate  
 Pi  
 Pow  
 Radians  
 Randint  
 Random  
 Randrange  
 Sample  
 Seed  
 Setstate  
 Shuffle  
 Sin  
 Sinh  
 Sqrt  
 Sum  
 Systemrandom  
 TENS  
 Tan  
 Tanh  
 Uniform  
 Vonmisesvariate  
 Weibullvariate  
 Wichmannhill  

ZEROS

\_1DimplicitSolveMethods

\_all\_complex

\_all\_float

\_all\_int

\_all\_numpy\_complex

\_all\_numpy\_float

\_all\_numpy\_int

\_avoid\_math\_symbols

\_classes

\_complex\_types

\_constants

\_float\_types

\_functions

\_implicitSolveMethods

\_indentstr

\_int\_types

\_localfuncnames

\_num\_equivtype

\_num\_maxmin

\_num\_name2equivtypes

\_num\_name2type

\_num\_type2name

\_num\_types

\_pytypefromtype

\_real\_types

\_seq\_types

a

acos

acosh

all  
 allmathnames  
 allmathnames\_symbolic  
 alphabet\_chars\_RE  
 any  

arccos

arccosh

arcsin

arcsinh

arctan

arctan2

arctanh

argmax  
 argmin  
 argsort  

argstr

asarray  

asin

asinh

atan

atan2

atanh

atleast\_1d  

builtinFnSigInfo

builtin\_auxnames

builtinlookup

c

ceil

clip  
 collect\_numbers  
 complex  
 complex128  
 complex192  
 complex64  
 complex\_  
 complexfloating  
 concatenate  
 convert\_power\_reserved\_keywords  

cos

cosh

double  

e

ensureints  

exp

f

fabs

feval\_map\_const

feval\_map\_symb

finfo  
 fit\_function  
 float  
 float32  
 float96  
 float\_  
 floating  

floor

fmod

fname

frexp

funcnames

funcstr

greater  

hypot

i

int  
 int0  
 int16  
 int64  
 int8  
 int\_  
 integer  
 inverseMathNameMap  
 isVectorClause  

isfinite

ldexp

less  
 less\_equal  

local\_fndef

log

log10

logical\_or  

m

mat  
 mathNameMap  

math\_dir

math\_globals

mathlookup

minpack  

mod

modf

modlookup

n

name\_chars\_RE

ndarray  
 newton\_meth  
 norm  
 npy  

null\_predicate

null\_predicate\_class  

num\_chars

ones  
 parser  

pi

pow

power

predicate\_op  

protected\_allnames

protected\_auxnamesDB

protected\_macronames

protected\_mathnames

protected\_randomnames

protected\_scipynames

protected\_specialfns

putmask  

qtypes

randomlookup

re  
 redirc  
 replaceSepQSpec  
 replaceSepStr  
 rerr  
 reserved\_keywords  
 rout  
 searchsorted  
 shape  
 sign  

sin

sinh

specTypes  
 specialfns  
 spy  

sqrt

swapaxes  

symb

symbol  

syms

take  

tan

tanh

targetLangs

temp\_macro\_names  
 temp\_macro\_names\_inv  
 time  
 toCircumflexSyntax  
 toDoubleStarSyntax  
 token  
 transpose  
 zeros  

---

[hide private]
